# “That would be such a game changer”: A qualitative study of the acceptability and feasibility of a digital harm reduction support tool among people who use drugs

**DOI:** 10.21203/rs.3.rs-7527847/v1

**Published:** 2025-10-14

**Authors:** David Frank, Raagini Jawa, Corona Zhang, Kelly Thompson, Thomas Pointer, Jennifer Muggeo, Tamar Krishnamurti, Scout LoPriore, Jess Tilley, Paul J. Joudrey

**Affiliations:** New York University; University of Pittsburgh; Ledge Light Health District; Alliance for Living; Yale School of Medicine; Ledge Light Health District; University of Pittsburgh; Alliance for Living; Harm Reduction Hedgehogs of 413; University of Pittsburgh

**Keywords:** harm reduction, decision aid, naloxone, drug checking

## Abstract

**Introduction:**

People who use drugs (PWUD) in the U.S. are often unable to effectively access social services and medical treatment including harm reduction services. A digital harm reduction support tool (DHRST) that would identify a client’s needs and match them to existing community services, is a potential strategy for reducing harms and optimizing access to preferred available services. We examined the feasibility and acceptability of a DHRST among PWUD and its potential for facilitating safer drug use practices and increasing engagement with social and medical services.

**Methods:**

We recruited a convenience sample of 37 clients from a community harm reduction service provider to participate in semi-structured interviews. Participants were required to be at least 18 years old; speak English; and be able to understand and provide informed consent. Interviews lasted approximately 30 minutes and focused primarily on individuals’ perspectives of a DHRST.

**Results:**

Most participants expressed enthusiasm for a DHRST. They described it as potentially filling a number of needs including providing information about local harm reduction services and as a platform to exchange information about the safety/quality of local drugs. Participants shared uncertainly about whether a DHRST was needed, fears about privacy, and perceived some risks of providing information about drugs.

**Conclusions:**

Although participants raised important concerns that would need to be addressed during DHRST development, findings were mostly supportive of DHRST acceptability and feasibility and underscored PWUD’s belief in its potential and willingness to use one.

## Introduction

Reducing overdose and other harms linked to substance use depends in part on the ability of people who use drugs (PWUD) to effectively access social services and medical treatment responsive to their individual needs. Yet, previous research shows that limited awareness and capacity can lead to underuse of some services and oversubscription of others (1–4). Information about services is highly local, often siloed and scattered, making it difficult for PWUD to access specific, usable information about their healthcare needs.

One strategy for improving access to services is to develop a patient/client decision aid. Decision aids are tools for providing tailored information to specific populations that help them make informed choices most aligned with their needs (5, 6). Often app-based, they have been successfully used in several healthcare settings including medication for opioid use disorder treatment settings and are shown to improve patients’ satisfaction, communication between patients and treatment providers, and to increase providers’ knowledge about their patients (7–9).

However, decision aids have not yet been adapted to address harm reduction services for PWUD that are not based on a need to stop using drugs or attend treatment (10, 11). Harm reduction organizations provide PWUD with equipment for safer drug consumption and naloxone (i.e., overdose reversal medication) and serve as a bridge between PWUD and more traditional social and medical services in their community (12, 13). Other creative efforts to increase access to harm reduction, such as vending machines that provide naloxone, sterile injection equipment, and other harm reduction tools have been successful and are associated with decreased overdose death and HIV incidence (14). Insufficient availability is not the only reason PWUD do not access harm reduction or other local services. Lack of opportunity to identify acceptable services that accommodate individual circumstances represents a key barrier to access. The current lack of an effective tool to help PWUD navigate the complex and evolving service environment may represent a missed opportunity to understand clients’ needs and facilitate greater engagement with services.

A digital harm reduction support tool (DHRST), informed by local knowledge and administered upon engagement with harm reduction services could help fill this gap and improve access to services for PWUD. A DHRST could identify clients’ preferred needs and match them to existing community services such as agencies that provide help with medical services, housing, employment, and legal issues. It could also serve as an information repository where PWUD could share information or reviews about local service providers and the local drug supply, including drug checking and alerts about particularly potent brands/stamps or clusters of overdoses. Moreover, a DHRST has the potential to enable systematic assessment of clients’ needs that could be used to inform advocacy and community resource planning as well as generate new knowledge about the medical and social needs of people engaging with harm reduction services.

Previous research also suggests the feasibility of adapting decision aids to a digital format. A recent study found that 96% of PWUD had mobile phones, and 92% had smartphones (15). Patients also reported currently using apps on their phone, including 40% who reported previously using any apps for mental health purposes (15).

While DHRST appear to hold promise, little is known about how acceptable and feasible a decision aid is for PWUD. To better understand this, we engaged in an exploratory assessment by conducting semi-structured interviews with PWUD. This article describes our findings.

## Methods

### Study setting and research team

Participants were recruited from Alliance for Living (AFL), a non-profit harm reduction service organization located in New London, CT, a small-to-medium-sized city (population approximately 27,000). AFL provides services for PWUD including a syringe service program, drug checking, infectious disease testing, and client navigation and connection to community medical and social services. AFL served over 1000 clients in 2023.

Our research team included a diversity of stakeholders, voices, and social positionalities including people from different racial, ethnic, and gender groups, as well as people with lived and living experience of drug use. Similarly, our study benefited from a strong community partnership with AFL and the inclusion of community organizational leaders in all aspects of the project. This study was approved by the New York University Washington Square Institutional Review Board (IRB-FY2023–7838).

### Sample and Recruitment

Our sample of 37 participants included PWUD who presented to AFL for harm reduction services. Participants were required to be at least 18 years old; speak English; and be able to understand and provide informed consent. We initially conducted 39 interviews. However, we excluded two interviews which were not recorded due to technical problems. Participants were recruited using a convenience sampling strategy in cooperation with AFL. Participants completed a survey assessment of demographics, housing status, and overdose history. The majority of participants completed the survey prior to being interviewed. However, due to the day-to-day realities of running a SEP, some participants were unable to complete the survey, or completed it after the interview portion of the study.

### Data collection

Interviews were conducted in a private office located in the back of the building and connected to a secure, remote meeting space (NYU’s secure Zoom application). In the interview, participants were first asked about their experiences using drugs and harm reduction services. Following this, they were given a brief description of a DHRST and asked to comment on its feasibility (“Do you think the tool I described would be an effective way of getting and receiving information about HR services; local drug quality, etc.?”; “What concerns do you have about the privacy of the information you share?”; “Is a phone-based app an effective way of obtaining healthcare information?”) and acceptability (“Do you think a tool like this would help you in your daily life? How?”) of the tool, both for themselves and for PWUD generally. For example, participants were asked if they” think the tool I described would be an effective way of getting and receiving information about HR services; local drug quality, etc.?”, The interview guide was developed collaboratively by the investigative team including AFL staff.

All interviews were audio recorded for later transcription; video was also recorded when participants expressed comfort with including video. Interviews lasted 30–45 minutes and participants received a $30 Walmart gift card for participating in the interview. All participants are referred to by pseudonyms.

Interviews were conducted using a situated approach based in part on the interviewer’s (Frank) lived/living experience using illegal opioids and in methadone maintenance treatment (MMT). Situated approaches are those that acknowledge the positionality and power dynamics existing between researcher, subject, and participant (16–18). As such, Dr. Frank disclosed his own status as a person who uses drugs and who is currently in MMT to participants. Since situated research places a greater emphasis on transparency, reflexivity, and social justice, than on neutrality and objectivity, scholars have noted the benefits of this approach for research that examines structurally and/or ideologically marginalized populations (19–21).

### Analysis

Interviews were analyzed using a thematic analysis strategy that sought to identify recurring themes in the data (22, 23). Data was coded on Atlas.ti by Frank using a combination of inductive and deductive approaches. Aprioi codes included: desirability of DHRST; uses for a DHRST; concerns about a DHRST; how information is shared in the local community; internet access; use of harm reduction services; unmet needs of PWUD; and others. Additional codes, such as “privacy concerns” were added based on analysis of the data. Reliability was assessed at multiple points in the analysis based on discussions with co-investigators (Joudrey, Thompson, Muggeo, Jawa, Krishnamurti) and in-line with practices established by similar qualitative, exploratory studies (24). For the thematic analysis, Data was organized into meaningful categories based on the aims of the study, the tenets and philosophy of harm reduction (25), and existing literature (22, 26, 27).

## Results

### Sample characteristics

Our sample included a total of 37 participants. 72% (n = 27) identified as cis men; 21% (n = 8) identified as cis women; and 5% (n = 2) either did not answer the question or identified as a gender not identified in the survey. Participants’ median age was 45 years old. 57% (n = 21) participants identified as “white”; 19% (n = 7) identified as “Latino/ a/ x”; 10% (n = 4) identified as “Black”; 5% (n = 2) identified as “Native American/Alaska Native”; 2% (n = 1) identified as “multiracial”; and 5% (n = 2) did not answer this question.

Participants described a wide variety of living situations. 30% (n = 11) stayed in their own home/apartment; 16% (n = 6) reported living with a friend or family; 19% (n = 7) were currently homeless or living “on the street”; and 13% (n = 5) were staying in a shelter at the time of the interview.

### People who expressed enthusiasm for a DHRST

Among the 37 interviews, 26 participants were enthusiastic about the prospect of a DHRST. Individuals described several potential uses for a DHRST; however, the ability to access peer-provided information about the local drug supply was the most cited advantage. For example, several participants voiced interest in using a tool to make safer choices about their drug use. They stated:

“That would be such a game changer because I’ve thought about something like that before and just, bro, here in New London, it’s not that big, but it is a small city, but so many people get bad batches and stuff. And me myself, I try to tell people, “Avoid this person, avoid this, avoid that,” because I’ve had a lot of people died on me, man.”

-Charles, 39-year-old white man

“That would be super helpful. I think if you’re doing drugs, there should be an app with all that type of stuff where you can get new rigs, [find out about] drugs you should stay away from, housing, all that.”

-Henry, 33-year old white man

“I mean to alert people, like you said, ‘don’t go this area’, or if they send alerts, ‘don’t buy these stamps or bags’ or such things like that. It would be useful.”

-Natalie, 33-year-old white woman

Some explained that the DHRST may be particularly useful for individuals with fewer social contacts, either because of geographic relocation or from decreased engagement with social networks where information about drug use is exchanged. For example, D20 said:

“I’m actually from the center of the state, which is more city-like, and then I moved out here. So, when I moved out here, I was surprised that there’s barely any resources like that, and I don’t know anybody out here either, so I had no word of mouth.”

-Jane, 38-year-old white woman

Participants also reported difficulty obtaining up to date information about local services available for PWUD and believed a DHRST could help. They pointed out that a DHRST could include information about the availability and intake procedures of drug treatment facilities as well as information about how to navigate local housing and employment services. They stated:

“I mean, it’s stuff like that [information on local drug supply], but also different detoxes, like detoxes of your area, services of that sort. Because, I mean, me, I’ve been trying to get into somewhere [for treatment] for three weeks, and it’s just come to the point of so discouraging that I’m ready to give up. I want to go and get clean, and I can’t.”

-Jackson, 38-year-old Native American/Alaskan man

“Any resource or any avenue towards recovery, reverse homelessness, or even feed someone, or even small things like gift cards and such. Those are things that can assist people. So any type of resource that’s available, I would think it’s good. Yeah. Because each resource could offer different things.”

-Dorthey, 60-year-old Black woman

They also noted that while information about local services does exist, it is often inconsistently organized, out of date, and difficult to access in a useful way. Participants suggested a DHRST could fill this gap by making timely information available online or through a cellphone application, and thus more accessible to PWUD. They argued:

“You can get that [information] at any shelter or any program run like this. But to have that access at your fingertips like that, I think it can go a long way.”

-Hector, 54-year-old LatinX man

“The information’s out there, it’s everywhere. But I think if it’s there on an app, it’s all in one spot and it’s in your hand, basically. You can get information from all different places, but you got to be at all those different places to get the information.”

-Laverne, 38-year-old white woman

Participants particularly emphasized the need for practical, often difficult to obtain information such as the dosing or take-home policies of local medication for opioid use disorder providers and whether local shelters allow alcohol and/or drug use. Participants saw this information as critical for determining if services are acceptable and appropriate for their needs. For example, participants reported:

“The detox is this many days. This is how they medicate you or don’t medicate you. Those are the things that’s really important… Shelters, are they dry? What time do they kick you out? It’s hard to get that information sometimes. You call the programs and they don’t really tell you too much.”

-Jane, 38-year-old white woman

“I’m on a MAT [medication assisted therapy] now, but before I was scared to go to detox because I didn’t know what it entailed, the steps, who I would have to talk to.”

-Jerome, 45-year-old Black man

Some participants compared the idea of a DHRST to currently available online sources of information about drugs such as Bluelight.com (i.e., an online, community-based forum where people with experience using drugs, can post about their knowledge and experience with substance use) or reddit. Yet they explained that such services were not as useful for local markets and rarely included the kinds of information that PWUD needed to make decisions about drug use or access to services. For example, they stated:

“[Sites like Bluelight], they don’t [get granular enough]. A lot of the moderators don’t really like them being very specific about the bags, that type stuff. Then resource-wise, they don’t steer you in any direction. They don’t say, “Hey, oh, there’s these, this program’s good. This one’s bad”. Nothing going on like that.”

-Jane, 38-year-old white woman

“I go on Reddit. You know what Reddit is? They’ve got a fentanyl sub-reddit, they got a heroin sub-reddit. But the thing is, it’s like you’re going through such a huge, you got people from all over the world type of shit, here or there, but it’s different stuff. All right, so in my area, there’s this purple rock fentanyl that’s starting to come around, but I knew it was going to come around, because people were posting it a few months ago in New York, Canada. And I knew it was coming here. So, when it came here, you know what I mean. Something more localized, centralized, that would be very helpful.”

-Doug, 45-year-old white man

Some also compared the idea to current efforts by harm reduction providers to inform local PWUD about the quality of local drugs. For example, some participants described the DHRST as a digital version of a whiteboard currently located in AFL that the local community uses to communicate important information about the local drug supply. As A18 explained:

“They try to do that here with their whiteboard. Yeah, they have a dry erase board in the SSP area, and you can write “The blue Fentanyl, it’s bad. It’s got xylazine in it.”

-Laverne, 38-year-old white woman

### Participants who expressed concerns about a DHRST

However, 11 participants said either ‘no’ or ‘maybe’ to the idea of a DHRST and expressed doubts about their willingness to use one. Among this group, privacy was the most-frequently mentioned concern. Participants had fears about how law enforcement could potentially use the DHRST to surveil the activities of PWUD, as well as about family members or friends seeing something the individual wanted to keep private. For example, participants stated:

“What I think should happen though is if you’re going to make an app like this and invite people to want to be comfortable, you’re going to have to either involve law enforcement in the sense of, ‘we don’t want you to have anything to do with this. We’re trying to save lives, not get people arrested.’ So, law enforcement would have to be completely removed if any of this is going to work.”

-Neil, 26-year-old white man

“I could see how somebody might not want to put something on there. Somebody sees it, and they’re like, “Oh. This is my mom,” or, “This is my brother,” or, “This is...” You know what I mean?”

-Doug, 45-year-old white man

“I don’t want to do nothing like that on my phone… Because I keep my business to myself.”

-Alan, 63-year-old white man

Yet, most participants stated that as long as the DHRST were held to the same privacy standards that govern other app-based products, they would feel comfortable using it. The following responses reflect this view:

“I mean, [I am] not really [concerned about privacy]. As far as, it’s going to be an app, so you’ll have the ability to give it permission on your phone if you don’t want to let it to be in every aspect of your phone, you can just say no.”

-Laverne, 38-year-old white woman

“I mean, as long as when I open the [app], it tells me what I’m consenting to and what type of information’s going out. That’s same with any app that I use.”

-Connie, 38-year-old white woman

Participants also worried about the potential unintended consequences, including violence, of making sensitive information about drug selling markets public. As D12 described:

“Because people buy drugs, on the streets, if you’re going to go to social media, as soon as one video [comes out], that person is a snake. So, they’re going to be watching, and they’re going to be watching people, people can get hurt. I don’t think that would be a good thing to bring out there, how to get drugs or where.”

-Jacob, 36-year-old white man

In addition to concerns about privacy and safety, some participants believed that drug use should be discouraged and worried that providing information about the quality and/or risks of particular drugs would facilitate at-risk drug use. For example, the following participants stated:

“I mean, I don’t really think so [that it’s a good idea], because then you have more people together which will associate with getting more drugs.”

-Alexander, 38-year-old Native American/Alaskan man

“Usually see people that hear about a bad batch of dope, they run to it.”

-Cole, 52-year-old white man

Lastly, some participants raised concerns about the need for a DHRST. They pointed out that in most drug using communities, people are familiar with one another and usually obtain information about drug use and related services through word of mouth. For example, they stated:

“I’ve been in the drug game a long time. I’ve never needed an [app] before.”

-Max, 49-year-old Black gender unidentified

“But really, do you need an app to do that? You could get referral services from a human person, and then go in person and find out more about it in person.”

-Nathaniel, 59-year-old white man

### Participants had ideas for using a DHRST in creative ways

Participants had a number of creative and thoughful ideas for maximizing the impact and utility of a DHRST. For example, some suggested including a means of sending alerts to PWUD about ‘bad batches’, or clusters of overdose in particular areas. Others described allowing PWUD to send an alert to emergency services if they believe they might be overdosing. For example, participants stated:

“You know how when you get an Amber alert, it beeps and it gives you the information. Well, if you have the app, I’m sure that they could have where the phones beep and it warns you of overdoses in the area - it warns you about what’s going around.”

-Dale, 28-year-old white woman

“They have those things for people with, I don’t know if it’s a kidney or liver or something like that. They wear around their neck, and you just hit the button. You hit a button. They should come up with a tool like that for people so if they feel like they’re fading out, you hit this button and next thing you know they’re on the way”.

-Kathlene, 34-year-old white woman

“Emergency notifications for tainted drugs would be awesome.”

-Shane, 38-year-old man, race unknown

One participant also suggested using photo ID technology to help PWUD identify the strength/quality of their drugs.

“You take a picture of the bag of the stamp and then people will tell you how that shit is. Or to be careful if it’s pretty strong.”

-Jerry, 34-year-old white man

Thus, participants were excited by the proposect of using this technology to improve safety and quality of life of PWUD and had multiple ideas about how a DHRST could be used creatively to facilitate those goals.

### Participants preferred a smartphone-based tool

Most participants felt that a cellphone application would be the most easily accessible format for a DHRST. Although a small number raised concerns about access to cellphones, most reported having one and that most PWUD–even when unstably housed–maintain regular cellphone access. Yet, most also agreed that making the information available in as many formats as possible would provide the most access. For example, participants stated:

“Of course [it would be helpful]. Because the phone is the most used thing, a part of a person’s life. No matter if you’re an addict or not, poor or not, you got a phone, so you can get the services... Everything is right there. Boom.”

-Dimitri, 38-year-old white man

“I would say actually [put it on] all three [websites; internet; apps], because somebody like me who I don’t have a phone, but I just recently got a tablet, in order to find out about it initially, I’ll probably have to read it somewhere. So, all three, actually, especially at my age, I’m not as computer savvy as other people will be, and I’m used to something in print.”

-Jerome, 45-year-old Black man

## Discussion

This article examines the feasibility and acceptability of a DHRST among PWUD. Our findings suggest that there is substantial interest in, and perceived need for, such a tool among PWUD while also noting concerns that would need to be proactively addressed before such a tool would be usable. The potential benefits of a DHRST included helping PWUD to make safer and more informed decisions about where and from whom to purchase drugs, and facilitating improved access to services like housing support, healthcare, and substance use disorder treatment by providing local knowledge on service availability and appropriateness. Participants’ focus on using a DHRST to increase the safety of active drug use likely speaks to level of concern among PWUD about the risks associated with current state of the heavily adulterated, unregulated drug supply. Results also suggest that a DHRST may be particularly useful to certain populations of PWUD such as people who are new to an area or older individuals who may not be as involved with the local network of PWUD.

Previous studies show that patient/client decision aids can improve PWUD’s access to information and agency in healthcare (28–30), demonstrating their potential in harm reduction settings. However, our findings show that the development and use of a DHRST is contingent on the ability of developers to overcome key challenges including privacy, access to smartphones and/or tablets, and concerns about dissemination of community knowledge and resources increasing at risk drug use. Participants in this study most frequently mentioned privacy as their main concern which was not unexpected and is a commonly cited issue in the use of other decision aids (31–33). Other patient decision aids have addressed this issue primary through technological solutions that improve data security. For example, a PtDA developed for HIV treatment prescription uses an advanced cryptographic technology to preserve the privacy of the patient records and the confidentiality of the clinicians’ decisions (34). Developers would also need to address the unique privacy needs of this population. As study participants described, information intended to promote safety, such as comments about an unreliable drug-seller or dangerous product, can lead to problems or even violence within the community and may need to be restricted or moderated in some way. Assessments of local access to smartphones and tablets would also need to be carried out, and partnerships with local stakeholders, particularly harm reduction organizations and local service providers, would need to be established. Other considerations, such as whether and to what extent to involve local law enforcement in the development of a DHRST are complex and impacted by concerns about privacy and safety as well as the current relations between PWUD and local police and should be the subject of further research.

Our findings also suggest that any effort to develop a DHRST should also include meaningful collaboration with community stakeholders and particularly PWUD. Our collaboration with AFL was an important strength of this research that enabled investigators to collect up-to-date data that reflects the real-world needs of the local community. Similarly, allowing PWUD to ‘brainstorm’ about how a DHRST could be used generated several valuable ideas that were not initially considered by the study team–such as an ‘emergency button’ allowing users to immediately request emergency services for an overdose. This illustrates the importance of meaningful involvement of PWUD at all stages of research and in the development of any services meant for that population.

This article should be seen in the context of several limitations. First, perceptions about the need for a DHRST may vary based on the community setting. Our sample was recruited from a small-to-mid-sized city where PWUD are more likely to be familiar with one another than in a large city, potentially impacting the need for such a tool. Similarly, as with all qualitative research, results can not necessarily be generalized to the larger population of people who use drugs. Lastly, while our research team was inclusive of harm reduction service providers, we did not interview service providers for PWUD, a perspective that likely differs from PWUDs. Additional research can help to better understand the needs of these specific groups.

## Conclusion

Despite these limitations, our results are supportive of the development of a DHRST. The need for increased access to safer ways of using drugs is great, and a DHRST would help to fill that gap while also potentially facilitating connections between PWUD and housing, employment, and healthcare service providers. As such, and in consideration of the risks that PWUD encounter–particularly from the unregulated, contaminated drug supply (35, 36)–our findings support the continued development of a DHRST and identify key barriers to successful develop from the PWUD perspective.

## Data Availability

The datasets generated and/or analyzed during the current study are not publicly available due concerns for participants’ privacy, but are available from the corresponding author on reasonable request.

## Highlights

- People who use drugs expressed enthusiasm for a digital harm reduction support tool and described it as filling a number of needs.
- Needs that could be fulfilled include providing information about the local drug supply and harm reduction services, and serving as a platform to exchange information affecting the local drug-using community.
- Some participants voiced uncertainty about whether a digital harm reduction support tool was needed, shared fears about privacy, and perceived providing information about drugs as dangerous

## References

[1] Krug A, Hildebrand M, Sun N. “We don’t need services. We have no problems”: exploring the experiences of young people who inject drugs in accessing harm reduction services. Journal of the International AIDS Society. 2015;18:19442. 25724510 10.7448/IAS.18.2.19442 PMC4344543

[2] Parker J, Jackson L, Dykeman M, Gahagan J, Karabanow J. Access to harm reduction services in Atlantic Canada: Implications for non-urban residents who inject drugs. Health & place. 2012;18(2):152–62. 21955638 10.1016/j.healthplace.2011.08.016

[3] Krawczyk N, Allen ST, Schneider KE, Solomon K, Shah H, Morris M, Intersecting substance use treatment and harm reduction services: exploring the characteristics and service needs of a community-based sample of people who use drugs. Harm reduction journal. 2022;19(1):1–10. 36002850 10.1186/s12954-022-00676-8 PMC9400571

[4] Frank D, Mateu-Gelabert P, Guarino H, Bennett A, Wendel T, Jessell L, Teper A. High risk and little knowledge: overdose experiences and knowledge among young adult nonmedical prescription opioid users. International Journal of Drug Policy. 2015;26(1):84–91. 25151334 10.1016/j.drugpo.2014.07.013 PMC4277710

[5] Stirling C, Lloyd B, Scott J, Abbey J, Croft T, Robinson A. A qualitative study of professional and client perspectives on information flows and decision aid use. BMC Medical Informatics and Decision Making. 2012;12:1–8. 22458734 10.1186/1472-6947-12-26 PMC3349513

[6] O’Connor AM, Fiset V, DeGrasse C, Graham ID, Evans W, Stacey D, Decision aids for patients considering options affecting cancer outcomes: evidence of efficacy and policy implications. JNCI Monographs. 1999;1999(25):67–80. 10.1093/oxfordjournals.jncimonographs.a024212 10854460

[7] Mooney LJ, Valdez J, Cousins SJ, Yoo C, Zhu Y, Hser Y-I. Patient decision aid for medication treatment for opioid use disorder (PtDA-MOUD): Rationale, methodology, and preliminary results. Journal of Substance Abuse Treatment. 2020;108:115–22. 31668516 10.1016/j.jsat.2019.08.006 PMC7397558

[8] Stacey D, Légaré F, Lewis K, Barry MJ, Bennett CL, Eden KB, Decision aids for people facing health treatment or screening decisions. Cochrane database of systematic reviews. 2017(4). 10.1002/14651858.CD001431.pub5 PMC6478132 28402085

[9] Guille C, Jones HE, Abuhamad A, Brady KT. Shared decision-making tool for treatment of perinatal opioid use disorder. Psychiatric Research and Clinical Practice. 2019;1(1):27–31. 36101566 10.1176/appi.prcp.20180004 PMC9176026

[10] Wodak A, McLeod L. The role of harm reduction in controlling HIV among injecting drug users. Aids. 2008;22:S81–S92. 10.1097/01.aids.0000327439.20914.33 PMC3329723 18641473

[11] Milaney K, Passi J, Zaretsky L, Liu T, O’Gorman CM, Hill L, Dutton D. Drug use, homelessness and health: responding to the opioid overdose crisis with housing and harm reduction services. Harm reduction journal. 2021;18:1–10. 34446034 10.1186/s12954-021-00539-8 PMC8394031

[12] Gleghorn A, Rosenbaum M, Garcia BA. Editors’ Introduction: Bridging the Gap in San Francisco, the Process of Integrating Harm Reduction and Traditional Substance Abuse Services. Taylor & Francis; 2001. p. 1–7. 10.1080/02791072.2001.10400461 11332995

[13] Bartram M. ‘It’s really about wellbeing’: A Canadian investigation of harm reduction as a bridge between mental health and addiction recovery. International Journal of Mental Health and Addiction. 2021;19(5):1497–510. 34790080 10.1007/s11469-020-00239-7 PMC8578070

[14] Arendt D. Expanding the accessibility of harm reduction services in the United States: measuring the impact of an automated harm reduction dispensing machine. Journal of the American Pharmacists Association. 2023;63(1):309–16. 36549931 10.1016/j.japh.2022.10.027 PMC9870941

[15] Hsu M, Martin B, Ahmed S, Torous J, Suzuki J. Smartphone ownership, smartphone utilization, and interest in using mental health apps to address substance use disorders: literature review and cross-sectional survey study across two sites. JMIR Formative Research. 2022;6(7):e38684. 35797102 10.2196/38684 PMC9305402

[16] Naples NA. Feminism and method: Ethnography, discourse analysis, and activist research: Psychology Press; 2003.

[17] Moradi B, Grzanka PR. Using intersectionality responsibly: Toward critical epistemology, structural analysis, and social justice activism. Journal of counseling psychology. 2017;64(5):500. 29048196 10.1037/cou0000203

[18] Graham LJ, editor Discourse analysis and the critical use of Foucault. The Australian Association of Research in Education Annual Conference; 2005.

[19] Haraway DJ. 1991. Situated knowledges: The science question in feminism and the privilege of partial perspective. Simians, cyborgs, and women: The reinvention of nature. 1988:1.

[20] DeVault ML. Talking back to sociology: Distinctive contributions of feminist methodology. Annual review of sociology. 1996;22(1):29–50.

[21] Powers P. The methodology of discourse analysis: Jones & Bartlett Learning; 2001.

[22] Vaismoradi M, Jones J, Turunen H, Snelgrove S. Theme development in qualitative content analysis and thematic analysis. 2016.

[23] Castleberry A, Nolen A. Thematic analysis of qualitative research data: Is it as easy as it sounds? Currents in pharmacy teaching and learning. 2018;10(6):807–15. 30025784 10.1016/j.cptl.2018.03.019

[24] Williams M, Moser T. The art of coding and thematic exploration in qualitative research. International Management Review. 2019;15(1):45–55.

[25] Coalition NHR. We Work for the Harm Reduction Movement 2024 [Available from: https://harmreduction.org/.

[26] Maguire M, Delahunt B. Doing a thematic analysis: A practical, step-by-step guide for learning and teaching scholars. All Ireland Journal of Higher Education. 2017;9(3).

[27] Fereday J, Muir-Cochrane E. Demonstrating rigor using thematic analysis: A hybrid approach of inductive and deductive coding and theme development. International journal of qualitative methods. 2006;5(1):80–92.

[28] Divall P, Camosso-Stefinovic J, Baker R. The use of personal digital assistants in clinical decision making by health care professionals: a systematic review. Health informatics journal. 2013;19(1):16–28. 23486823 10.1177/1460458212446761

[29] Schott SL, Berkowitz J, Dodge SE, Petersen CL, Saunders CH, Sobti NK, Personalized, electronic health record–integrated decision aid for stroke prevention in atrial fibrillation: a small cluster randomized trial and qualitative analysis of efficacy and acceptability. Circulation: Cardiovascular Quality and Outcomes. 2021;14(6):e007329. 34107740 10.1161/CIRCOUTCOMES.120.007329

[30] Heen AF, Vandvik PO, Brandt L, Achille F, Guyatt GH, Akl EA, Decision aids linked to evidence summaries and clinical practice guidelines: results from user-testing in clinical encounters. BMC Medical Informatics and Decision Making. 2021;21(1):202. 34187484 10.1186/s12911-021-01541-7 PMC8240084

[31] Liu X, Lu R, Ma J, Chen L, Qin B. Privacy-preserving patient-centric clinical decision support system on naive Bayesian classification. IEEE journal of biomedical and health informatics. 2015;20(2):655–68. 10.1109/JBHI.2015.2407157 26960216

[32] Spini G, Mancini E, Attema T, Abspoel M, de Gier J, Fehr S, New approach to privacy-preserving clinical decision support systems for HIV treatment. Journal of medical systems. 2022;46(12):84. 36261621 10.1007/s10916-022-01851-x PMC9581834

[33] Tracy CS, Dantas GC, Upshur RE. Feasibility of a patient decision aid regarding disclosure of personal health information: qualitative evaluation of the Health Care Information Directive. BMC Medical Informatics and Decision Making. 2004;4:1–7. 15361257 10.1186/1472-6947-4-13 PMC518970

[34] Wimmer H, Yoon VY, Sugumaran V. A multi-agent system to support evidence based medicine and clinical decision making via data sharing and data privacy. Decision Support Systems. 2016;88:51–66.

[35] Quijano T, Crowell J, Eggert K, Clark K, Alexander M, Grau L, Heimer R. Xylazine in the drug supply: emerging threats and lessons learned in areas with high levels of adulteration. International Journal of Drug Policy. 2023;120:104154. 37574646 10.1016/j.drugpo.2023.104154

[36] Montero F, Bourgois P, Friedman J. Potency-enhancing synthetics in the drug overdose epidemic: xylazine (“tranq”), fentanyl, methamphetamine, and the displacement of heroin in Philadelphia and Tijuana. Journal of illicit economies and development. 2022;4(2):204. 37009634 10.31389/jied.122 PMC10065983

